# Recurrent Urinary Tract Infection in a Patient With Asymptomatic Crohn's Disease

**DOI:** 10.7759/cureus.9962

**Published:** 2020-08-23

**Authors:** Waqar Akram, Sanket K Shah, Mohina Sohail, Usama Rehman, Mustafa Rahim

**Affiliations:** 1 Internal Medicine, Raleigh General Hospital, Beckley, USA; 2 Anesthesia, Mayo Hospital, Lahore, PAK; 3 Internal Medicine, West Virginia University School of Medicine, Morgantown, USA

**Keywords:** urinary tract infection, crohn's disease

## Abstract

Recurrent urinary tract infection (UTI) is uncommon in males, but it is a common complication of Crohn’s disease (CD). Patients with CD often present with abdominal pain, diarrhea, and systemic symptoms, such as weight loss, low-grade fever, and fatigue, and rarely it can cause serious complications, such as fistulas or abscesses. Some patients with CD remain asymptomatic, which can progress to severe complications and delayed treatment. We are presenting a 22-year-old male with recurrent UTIs and no established past medical history of CD. However, on subsequent investigations, an anatomical abnormality was discovered that helped us make the diagnosis of CD. The aim of this report is to emphasize the early detection of asymptomatic CD in atypical patients, to not only decrease the risk of complications such as UTIs but also allow for early treatment intervention and better outcomes.

## Introduction

Urinary tract infection (UTI) is uncommon in male patients younger than 50 years. The causes of UTI include prostatitis, epididymitis, orchitis, pyelonephritis, cystitis, arthritis, and urinary catheters. Many experts believe that UTI in males is associated with anatomic abnormalities and might require surgical procedures to avoid further complications. Obtaining a urine culture in a suspected male UTI can help narrow down the diagnosis. In a majority of males with their first UTI, imaging adds little to supplement a careful history and physical exam. Men below 45 years of age with their first UTI who respond well to antibiotic treatment are not likely to have a urologic abnormality ‎[1,2].

An estimated 1.3% of adults in the United States are diagnosed with inflammatory bowel disease (IBD) either with CD or ulcerative colitis (UC). The risk of CD slightly increases in women and an Ashkenazi Jewish compared to non-Jews [2]. Age above 45 years, living below poverty, sedentary lifestyle, and unemployment are some of the commonly reported risk factors. Patients with CD often present with abdominal pain and diarrhea and may develop systemic symptoms, such as weight loss, low-grade fever, and fatigue. Fistulas or abscesses can develop in a patient with CD. Patient symptoms associated with the location of fistulae such as enterovesicular or enterourethral fistula present with recurrent UTI, enterovaginal fistula form passage of stool, enterocutaneous fistula cause drainage through the skin, and enteroenteric fistula cause diarrhea which can be bloody in severe cases [2,3]. Our aim in this report is to stress the importance of taking into consideration asymptomatic CD and pursing early detection in atypical patients presenting with recurrent UTIs. This will allow for early treatment intervention that will lead to decreased risk of complications and improved outcomes.

## Case presentation

A 22-year-old male patient who came to the hospital with recurrent UTI and hematuria complains of nausea, vomiting, flank pain, increased frequency of urination, cloudy urine, dysuria, and intermittent blood in the stool. However, he denied fatigue, fever or chills, abdominal pain, weight loss, constipation, diarrhea, urethral discharge, and pain radiating to the groin. The patient had no known medical or family history. The patient is a non-smoker, is sexually active with intermittent protection usage, and drinks alcohol occasionally.

**Figure 1:**
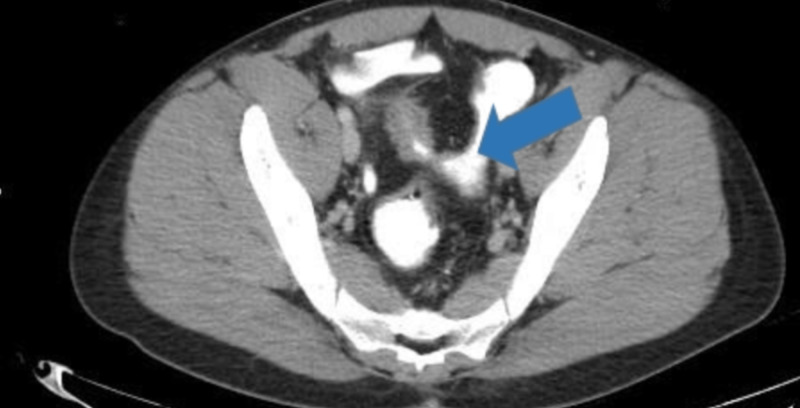
CT scan showing distal ileum with pseudodiverticulum thickened wall indicated by the blue arrow

On initial assessment, it appeared to be pyelonephritis and sexually transmitted infection (STI). However, STI was ruled out by history and lab tests. Imaging using CT and CT scan with contrast showed fistulas and the presence of an enterovesicular fistula. Axial CT scan revealed thickened pseudodiverticulum wall suggesting fistula (Figure 1).

**Figure 2:**
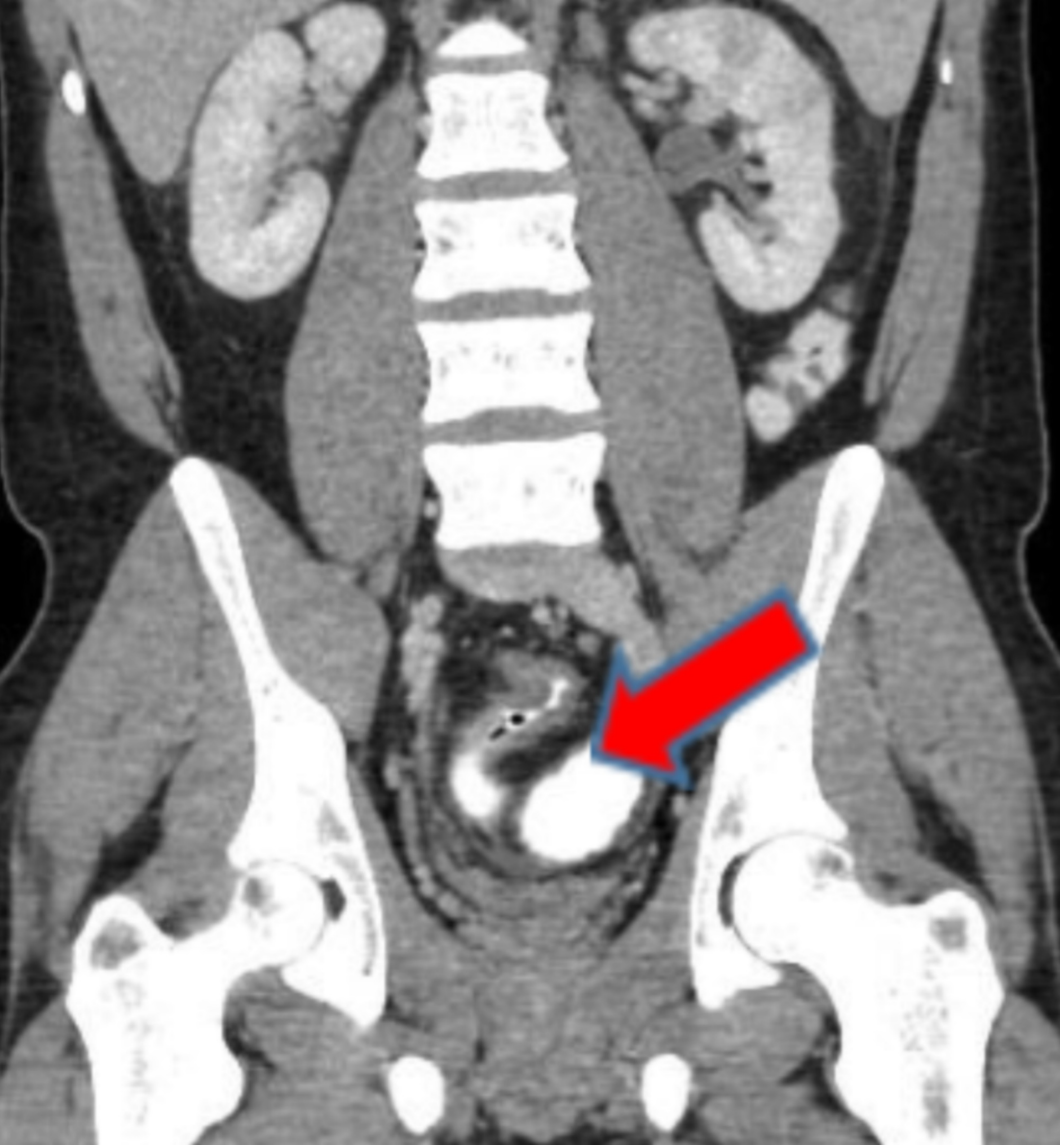
CT scan showing abnormal wall thickening of sigmoid colon indicated by the red arrow

Coronal scan of the abdomen shows thickened sigmoid colon suggesting chronic inflammation by bowel disease (Figure 2). 

**Figure 3:**
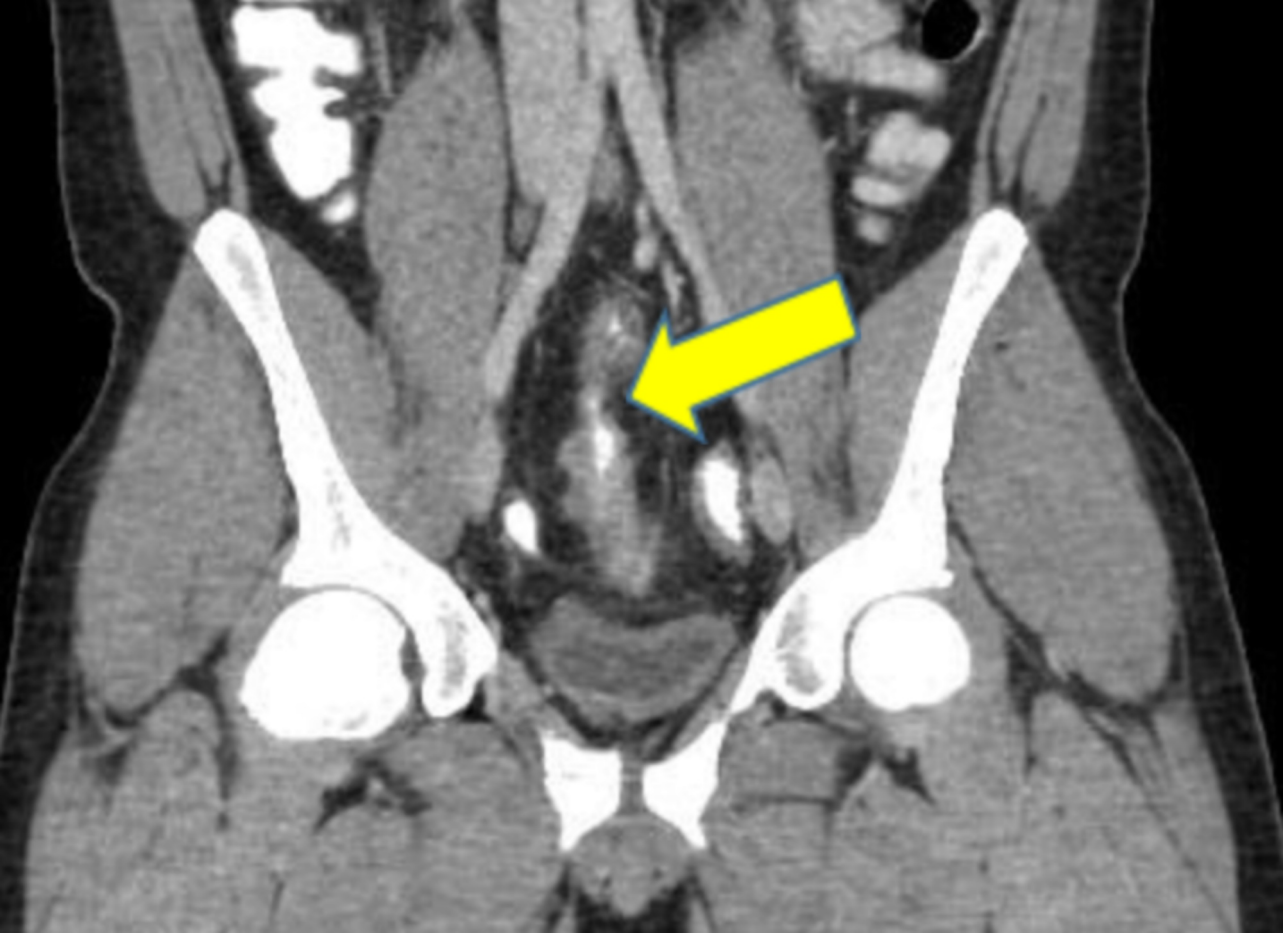
CT scan showing fistula between terminal ileum and sigmoid colon indicated by the yellow arrow

**Figure 4:**
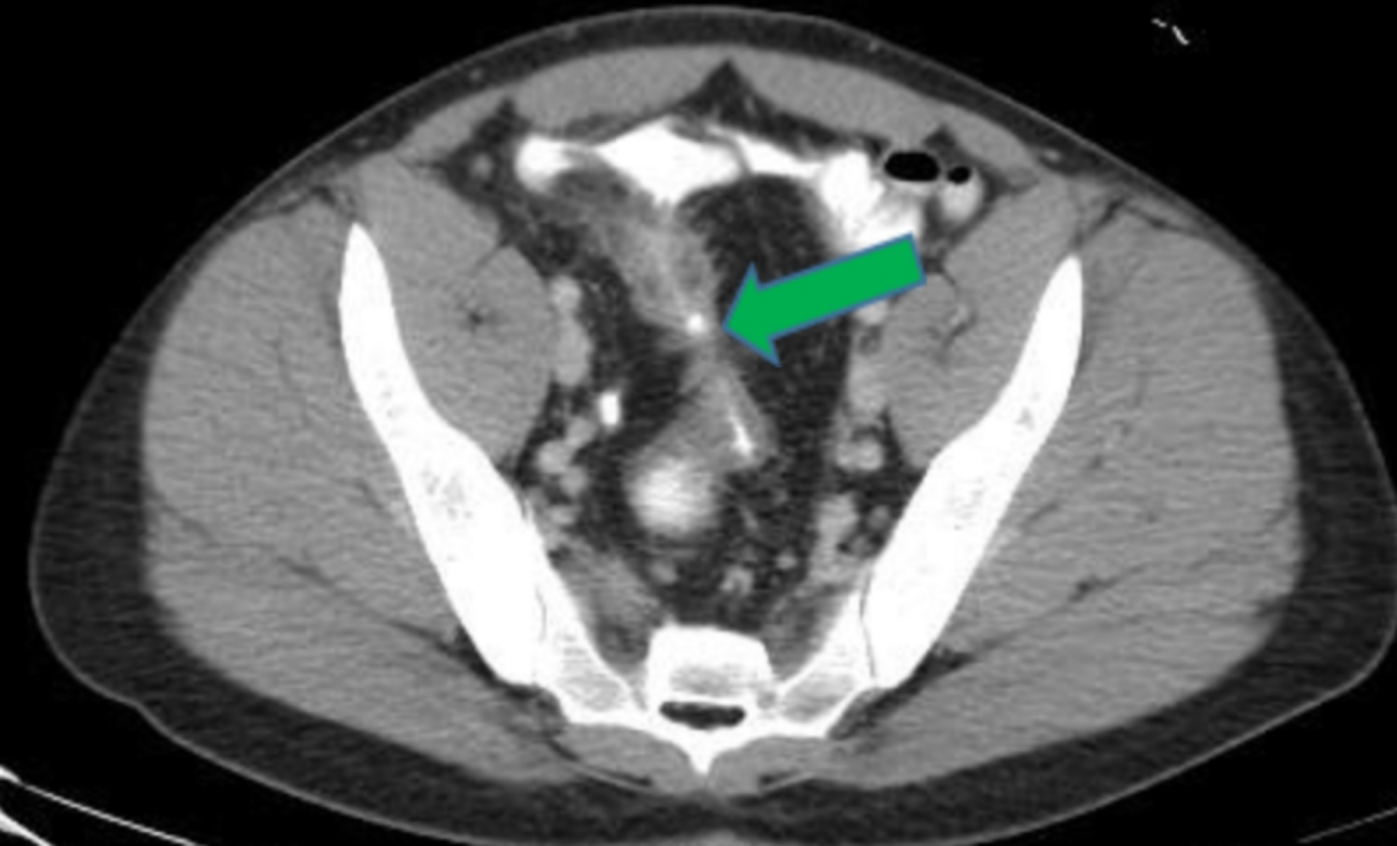
CT scan showing fistula connecting bowels indicated by the green arrow

Fistulas between small and large bowel suggest colonic fistula on CT scan (Figures 3, 4). 

**Figure 5:**
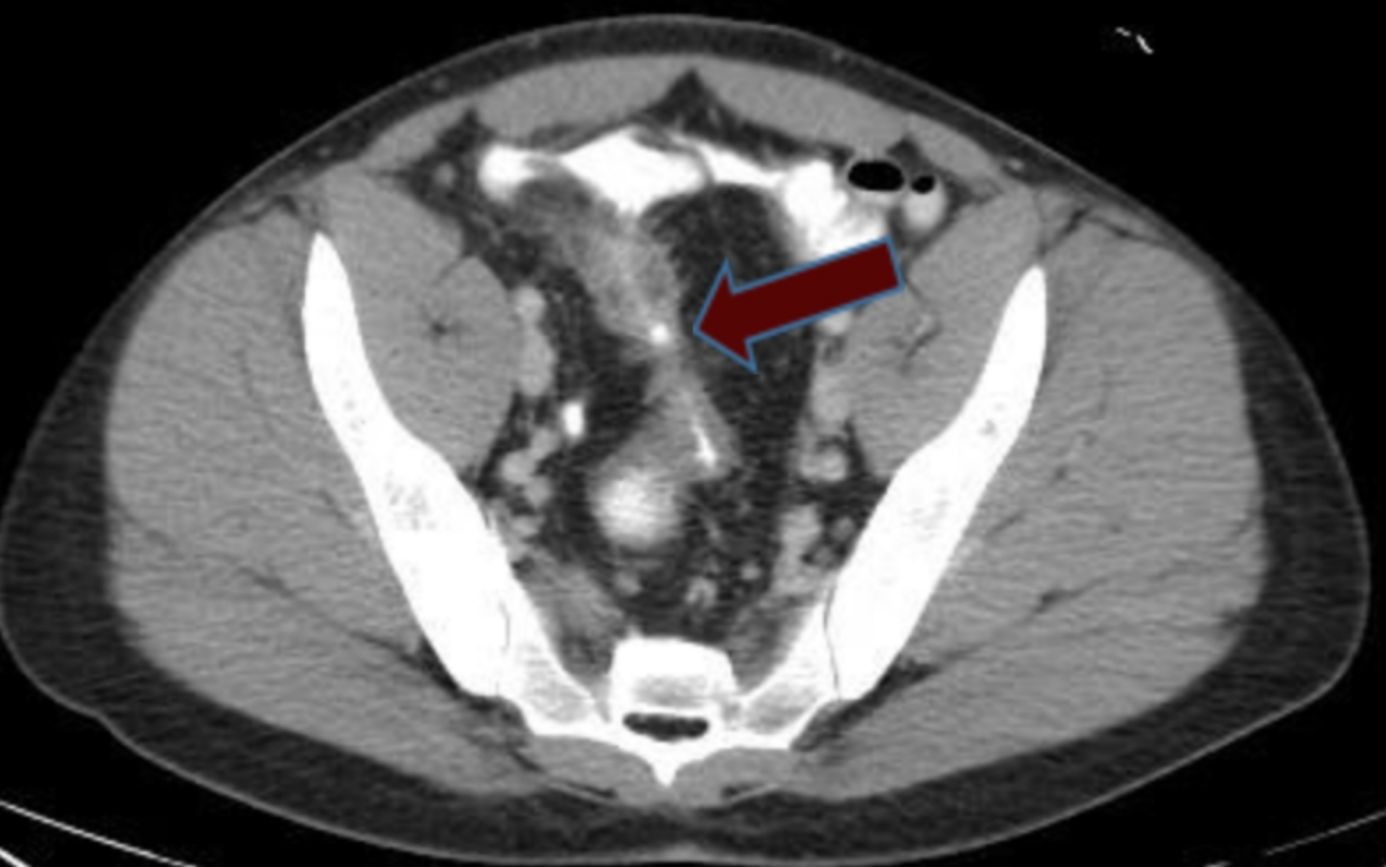
CT scan showing presence of fistula between the bladder and terminal Ileum indicated by the red arrow

Axial CT scan also showed the presence of a fistula between the bladder and the terminal ileum suggesting enterovesicular fistula (Figure 5). 

**Figure 6:**
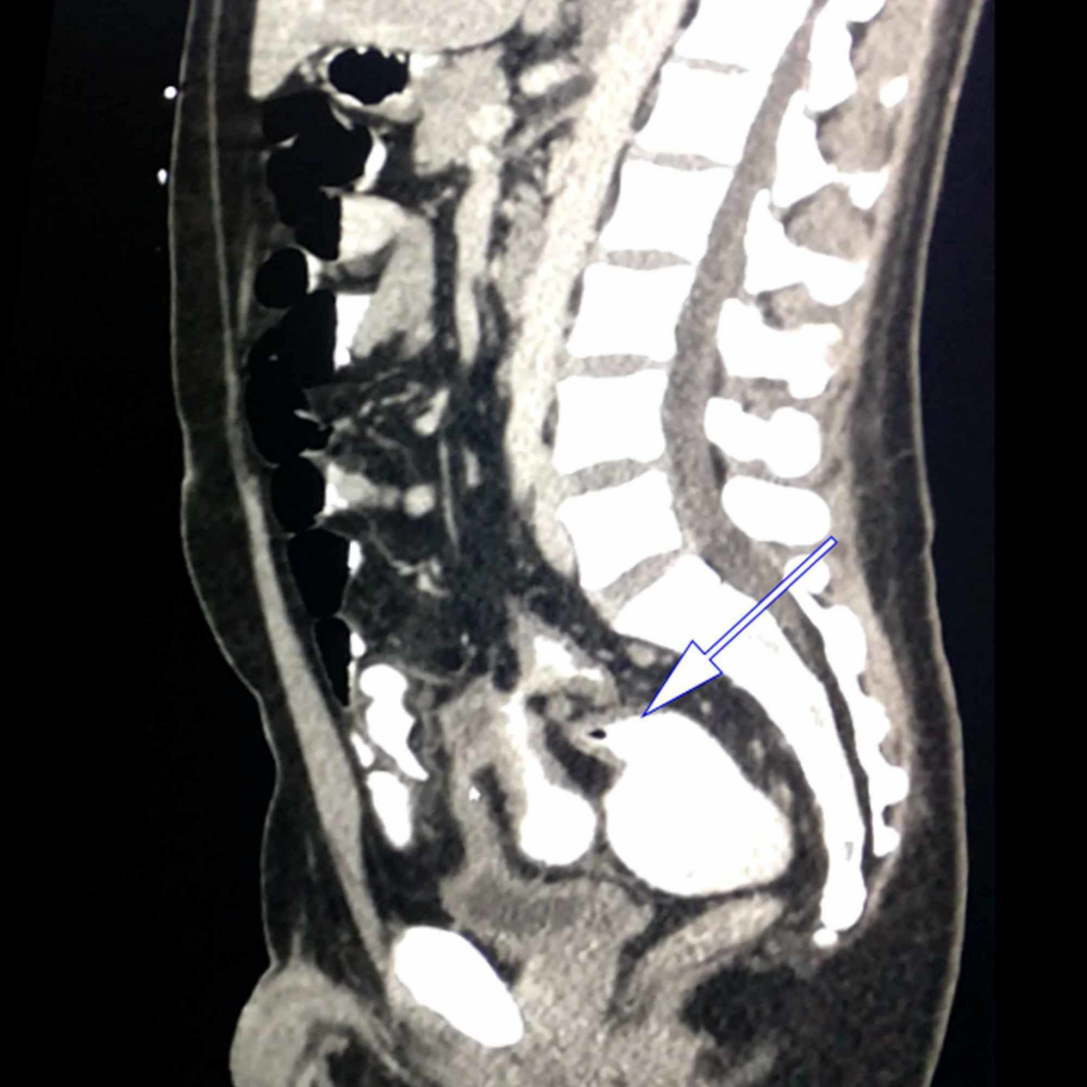
CT scan showing thickened sigmoid colon indicated by the white arrow

Sagittal CT scan shows the presence of thickened sigmoid colon suggesting chronic inflammation (Figure 6). 

**Figure 7:**
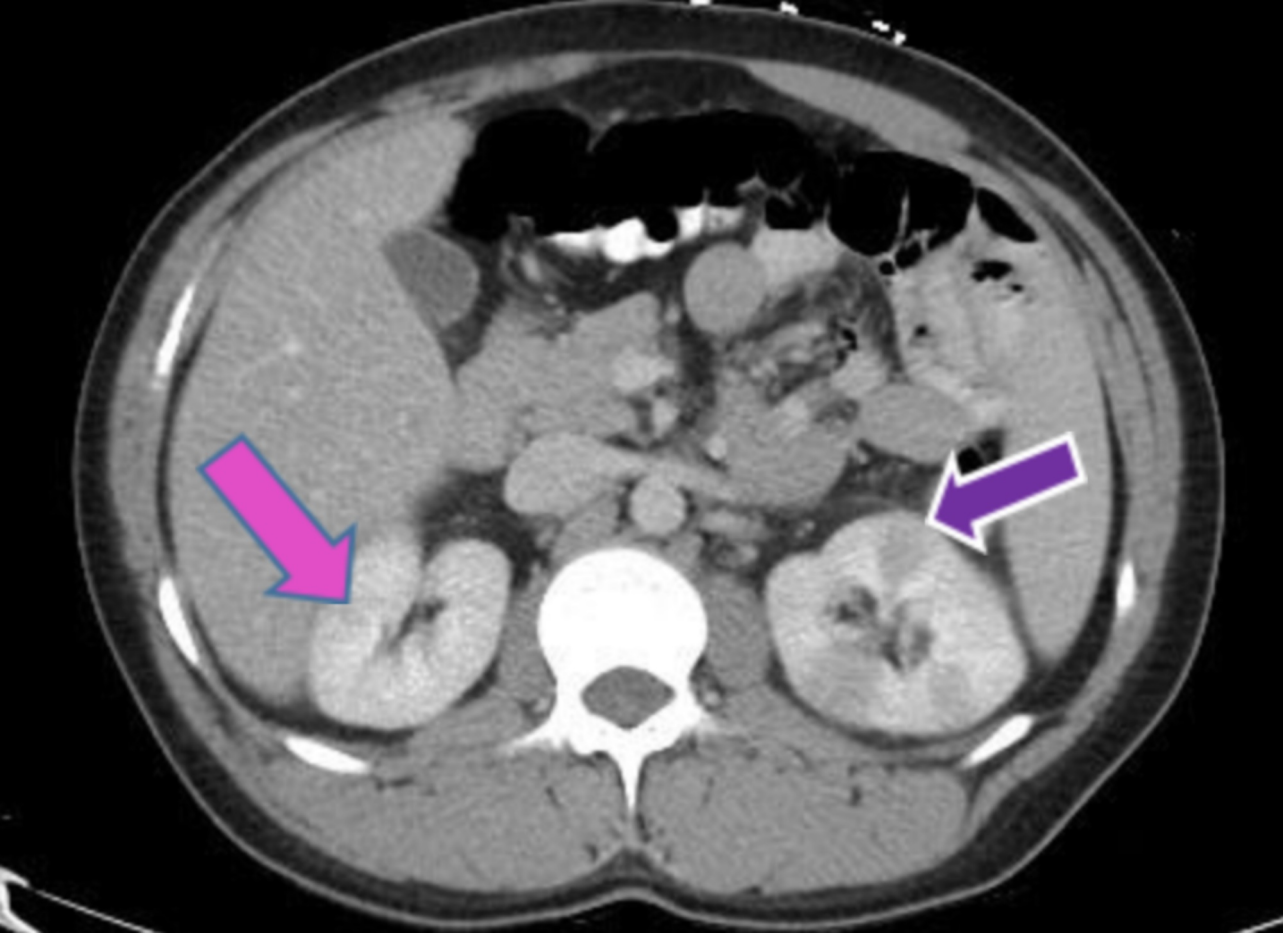
CT scan showing darkening of kidneys shows pyelonephritis indicated by the pink and purple arrows

The presence of hypodensity on a bilateral kidney CT scan is suggestive of pyelonephritis. Based on the CT findings and the presence of fistulas, the patient was diagnosed with CD (Figure 7). 

## Discussion

An incidence of UTI in males is five to eight cases per year per 10,000 cases with ages between 21 and 50 years, and prevalence is about 14%. Patients diagnosed with first UTI, with high-risk factors such as immunocompromised status, uncircumcised, engaging in anal intercourse, greater than 65 years of age, bladder outlet obstruction, anatomical functional abnormalities, previous urinary tract surgery, cystoscopy, catheterization, or transrectal prostate biopsy, may not need further unnecessary evaluation. Limiting further evaluation of men with a first UTI to those at increased risk may reduce unnecessary radiological, endoscopic, or urodynamic investigation. There is minimal evidence to support routine imaging in low-risk men with a first UTI, whether with or without fever [1,2].

Recurrent UTI in men warrants evaluation for structural abnormalities, colovesical fistulas associated with colonic malignancy or IBD, enlarged prostate, and unprotected sexual intercourse. Screening for immunologic disorders that might increase the risk of infection with the human immunodeficiency virus and hematologic malignancies is also recommended [3].

Organisms causing UTI can suggest the etiology. Gram-negatives such as Escherichia coli, Klebsiella pneumoniae, Pseudomonas aeruginosa, and Proteus mirabilis are often found in anatomical obstruction. On the other hand, organisms such as Chlamydia trachomatis, Neisseria gonorrhoeae, Trichomonas vaginalis, or Ureaplasma urealyticum are typically STIs. Yeast infections suggest underlying immunosuppression that occurs with diabetes or corticosteroid use [3].

CD follows a bimodal age distribution, with the first peak between the ages 15 and 30 years. Clinically, CD presents with abdominal pain, reduced appetite, diarrhea with blood in the stool, and weight loss. In patients who present with recurrent UTIs and are asymptomatic, physicians should always keep CD in mind, imaging techniques like CT scan and MRI for the extent of fistulas should be used and they decrease the high morbidity associated with it [1,4].

The European Crohn's and Colitis Organization (ECCO) and the European Society of Gastrointestinal and Abdominal Radiology (ESGAR) published new imaging guidelines for CD. The diagnosis of CD and UC requires multiple steps, which include clinical evaluation, stool sampling, endoscopy, imaging study, and histological findings. Multiple imaging studies help to establish diagnoses such as MRI, CT scan, magnetic resonance enterography (MRE), endoscopy, and colonoscopy. MRI is the first line study used to diagnose and classify perianal CD and provide a more accurate assessment of abscess and pelvic fistula. Transrectal ultrasounds are better than clinical exam in providing better information. A combination of MRI and transrectal ultrasounds provides a more accurate assessment [1,5]

Abdominal ultrasound, MRE, or a small bowel capsule endoscopy (SBCE) helps evaluate to newly diagnose a patient with CD and can be used in patients with negative endoscopy. Biopsies of both inflamed and non-inflamed segments are required to establish an accurate diagnosis. Patients with high clinical suspicion of CD and normal biopsy should be considered for SBCE or cross-sectional imaging. The presence of at least three small bowel ulcers on SBCE in a patient who has not used non-steroidal anti-inflammatory drugs for one month has a high chance of having CD [1,6].

The diagnostic studies, such as CT enterography and MRE, provide information that helps to treat a patient based on the severity and extent of CD and helps to evaluate its complications that cannot be observed by clinical exam or endoscopic evaluation of kids and adults. Based on the severity of the presentation, CD requires medical management or surgical intervention. For enterovesical fistula due to CD, medical therapy is the first choice. Zhang et al. reported long-term remission of enterovesical fistula in 13 of 37 patients with CD over a mean of 4.7 years through treatment with antibiotics, azathioprine, steroids, and infliximab [3,7-10].

Antibiotic therapy should be based on the organism and sensitivities when available. Fluoroquinolone and trimethoprim-sulfamethoxazole are first-line agents. Ustekinumab is a monoclonal antibody to the p40 subunit of interleukin-12 and interleukin-23 used in a patient with moderate to severely active CD. Research by Feagan et al. found that ustekinumab provides systemic anti-inflammatory activity, better safety profile, and has a lack of immunogenicity [11]. It is administered as one single dose intravenously, and subsequent use of subcutaneous injections provides a home-based advantage. It is more beneficial for a patient with the extraintestinal manifestation of disease and decreases the risk of infection. Ustekinumab had a higher response rate compared to placebo and used as maintenance therapy [3,5,8-11].

Definite treatment of enterovesical fistula is the surgical resection of the involved bowel. Gaglani et al. concluded that laparoscopic resection of IBD decreases hospital stays and operating time along with decreased complications [7]. It can offer a safer treatment possibility for IBD-associated fibrotic stricture. There is no need to repair the bladder wall because the vesical portion heals spontaneously. A patient who denies surgical repair can be managed by medical therapy [3,11].

## Conclusions

The initial workup for UTI consists of urine analysis and culture, but does not include imaging as it does not add diagnostic value. However, in atypical patients presenting with recurrent UTIs, after ruling out common etiologies, we recommend clinicians consider asymptomatic CD leading to an enterovesicular fistula. Extensive investigation using imaging techniques like X-ray, CT scan, MRI, MRE, and SBCE can be useful in establishing a diagnosis of CD. Pursuing early detection of asymptomatic CD in such atypical patients will allow for early intervention that can help reduce severe complications and improve outcomes.
